# Antimicrobial Susceptibility and Characterization of Extended-Spectrum β-Lactamases in *Escherichia coli* Isolated from Buffalo Mastitis Milk in Guangdong Province, China

**DOI:** 10.3390/microorganisms14051055

**Published:** 2026-05-08

**Authors:** Yunchen Zhou, Rong Xi, Siran Wang, Ban Li, Yue Wu, Chengbo Wen, Dexian Zhang

**Affiliations:** School of Animal Science and Technology, Foshan University, Foshan 528231, China; zhouyunchen2025@126.com (Y.Z.); 17866927857@163.com (R.X.); siran325@163.com (S.W.); a13138288071@163.com (B.L.); wuyue12262022@163.com (Y.W.); bwcheng0023@163.com (C.W.)

**Keywords:** ESBL-producing *E. coli*, buffalo mastitis milk, AMR, MLST, virulence genes

## Abstract

Antimicrobial resistance (AMR) in *Escherichia coli* (*E. coli*) from food-producing animals constitutes a substantial public health concern. This study characterized antimicrobial resistance profiles, phylogenetic diversity, virulence-gene distribution, and plasmid-borne extended-spectrum β-lactamase (ESBL) determinants of *E. coli* isolates recovered from water buffaloes with subclinical mastitis. Among the 54 ESBL-producing *E. coli* isolates, all were resistant to ampicillin and cefotaxime. High resistance rates were also observed for cephalothin (75.9%), trimethoprim–sulfamethoxazole (74.0%), ceftiofur (70.4%), florfenicol (68.5%), and cefazolin (63.0%). Lower resistance was recorded for colistin sulfate (40.7%), enrofloxacin (33.3%), and gentamicin (25.9%). Phylogenetic analysis of ESBL producers identified phylogroup B1 (42.6%) as predominant, followed by groups A (29.6%) and D (25.9%). Multilocus sequence typing (MLST) revealed that ST50 (20.4%) was the most common sequence type, and serogroup O150 was dominant (70.4%). Virulence genes, such as *iss* (81.5%), *astA* (59.3%), and *espP* (38.9%), were frequently detected among ESBL isolates. ESBL genes were predominantly *bla_CTX-M-1_* (27.8%) in all isolates, while the narrow-spectrum β-lactamase genes *bla_TEM-1_* (55.6%) and *bla_OXA-10_* (14.8%) were also commonly co-detected. Bioinformatic analysis predicted that all ESBL genes were associated with plasmid-derived contigs, with the predicted plasmid size ranging from approximately 32 to 187 kb and belonging to IncFIB, IncFIA, IncI1, IncFIA + I1, and IncFII replicon types. Conjugation frequencies ranged from 4.8 × 10^−7^ to 4.1 × 10^−2^, and plasmids were predicted to carry additional resistance genes mediating resistance to chloramphenicol (*floR*), sulfonamides (*sul1*, *sul3*), tetracyclines (*tet(A)* and *tet(B)*), and trimethoprim (*dfrA1*, *dfrA12*). The co-carriage of ESBL genes with additional antimicrobial resistance and virulence determinants suggests the potential role of water buffaloes as reservoirs of clinically relevant resistance traits that may disseminate through horizontal gene transfer.

## 1. Introduction

Antimicrobial resistance (AMR) poses a serious threat to public health. By 2050, AMR could cause approximately 10 million deaths annually and result in economic losses of around $100 trillion [1]. The misuse and overuse of antimicrobial agents in clinical practice have led to the emergence of diverse antimicrobial-resistant bacteria, including extended-spectrum β-lactamase (ESBL)-producing Enterobacteriaceae, carbapenem-resistant Enterobacteriaceae (CRE), vancomycin-resistant Enterococci (VRE), and methicillin-resistant *Staphylococcus aureus* (MRSA) [2]. Novel antimicrobial resistance mechanisms have raised increasing concern, such as the mobile colistin resistance gene (*mcr-1*) identified in pigs [3] and the plasmid-mediated tigecycline resistance gene *tet*(*X*) detected in pigs and chickens [4,5]. Notably, these resistance mechanisms are frequently reported in animals, especially food-producing animals. Consequently, they are considered not only reservoirs of antimicrobial resistance genes (ARGs) and antimicrobial-resistant bacteria (ARB) but also sources of novel resistance mechanisms [6]. Accordingly, products derived from food-producing animals may serve as potential vehicles for transmitting these ARGs or antimicrobial-resistant bacteria (ARB) to humans [7]. Therefore, increased attention should be paid to ARGs and ARB in food animals.

Among antimicrobial resistance mechanisms, ESBL production has become one of the most concerning forms of resistance to β-lactams, as it can inactivate oxyimino-β-lactams such as third-generation cephalosporins and aztreonam [8]. Moreover, ESBL-encoding genes, including *bla_SHV_* and *bla_CTX-M_*, are typically located on transmissible plasmids and can be acquired by susceptible bacteria through conjugation [9]. As a result, these genes have been isolated from humans, the environment, and animals in numerous countries, including China, and the dissemination of ESBL-producing isolates has become a serious public health concern [6]. Furthermore, ESBL-producing isolates are often resistant not only to β-lactams but also to fluoroquinolones, aminoglycosides, and sulfonamides, which are commonly used to treat or prevent infections in livestock [10]. ESBL-encoding genes are frequently linked to plasmid-mediated quinolone resistance, and *E. coli* isolates co-expressing plasmid-mediated quinolone resistance (PMQR) and ESBL can complicate treatment [11].

Buffalo milk is preferred by consumers in much of Southeast Asia because of its taste and quality. However, mastitis is the most prevalent disease in buffaloes, and various bacteria contribute to its occurrence, including *E. coli* and *Staphylococcus aureus* (*S. aureus*). Buffaloes with mastitis are recognized as reservoirs of ESBL-producing *E. coli*, and zoonotic transmission of such pathogens to animal handlers and consumers is possible. Owing to antimicrobial resistance, ESBL-producing *E. coli* often leads to infections that cause economic losses, increased veterinary and labor costs, and higher culling rates [12]. Moreover, it also poses a substantial threat to human health [13]. Therefore, investigating the virulence and antimicrobial resistance of ESBL-producing *E. coli* is of great importance.

Buffalo milk is popular in Guangdong Province, China, and mastitis has been extensively reported in this species. However, few studies have investigated the distribution of virulence genes and antimicrobial resistance in ESBL-producing *E. coli* isolates from buffaloes with mastitis. Our findings will provide information to guide antibiotic use when treating mastitis in buffaloes.

## 2. Materials and Methods

### 2.1. Sample Collection

Milking was performed twice daily on all farms. Sample collection involved forestripping (3–5 squirts), pre-milking teat disinfection with 0.25% iodine, and drying with a clean towel. After automatic cluster removal, post-milking teat disinfection with 0.5% iodine was applied. Duplicate quarter milk samples were aseptically collected following National Mastitis Council protocols [14]. Briefly, after discarding the first 3 streams, 3 mL of milk was collected from each quarter, stored on ice, and transported to the laboratory within 6 h. Subclinical mastitis was presumptively identified using a commercial California Mastitis Test kit (ImmuCell, Portland, ME, USA) according to the manufacturer’s instructions: 2 mL of milk was mixed with an equal volume of CMT reagent and stirred for 30 s, with gel formation indicating elevated somatic cell counts. CMT-positive samples were subsequently subjected to bacterial isolation and identification.

### 2.2. Isolation of E. coli

Isolation of *E. coli* was performed as described in our previous work [15]. Briefly, for *E. coli* isolation, a 0.1 mL milk sample was inoculated into 3 mL Mueller–Hinton broth (MHB; Oxoid, Shanghai, China), which was incubated at 37 °C for 24 h. Samples were then streaked onto MacConkey agar (AoBOX, Beijing, China) plates, which were kept at 37 °C for 24 h. Pink colonies were presumptively identified as *E. coli* and then subjected to matrix-assisted laser desorption ionization–time-of-flight mass spectrometry (MALDI-TOF MS) using a Microflex LT instrument (Bruker Daltonics, Bremen, Germany).

### 2.3. Identification of ESBL-Producing E. coli Isolates

All the *E. coli* isolates were screened for ESBL production using the disk diffusion method according to CLSI guidelines [16]. Isolates showing inhibition zone diameters of ≤27 mm for cefotaxime and ≤22 mm for ceftazidime were considered suspected ESBL producers.

Suspected isolates were further tested using a phenotypic confirmatory test. Briefly, isolates were spread onto Mueller–Hinton agar (MHA, Oxoid, Shanghai, China), and commercial antibiotic disks (ThermoFisher, Shanghai, China) containing ceftazidime (30 µg), ceftazidime–clavulanic acid (30/10 µg), cefotaxime (30 µg), and cefotaxime–clavulanic acid (30/10 µg) were placed onto plates. Inhibition zones were measured, and any isolate exhibiting a ≥5 mm increase in zone diameter for either antimicrobial agent tested in combination with clavulanic acid compared to the agent alone was confirmed as an ESBL-producing *E. coli* strain.

### 2.4. Antimicrobial Susceptibility Testing

Antimicrobial susceptibility testing was performed using the broth microdilution method in MHB (Oxoid) according to the guidelines of CLSI [16]. A loopful of each *E. coli* isolate preserved in glycerinated MHB (Oxoid) was streaked on MHA (Oxoid) and incubated at 37 °C for 24 h. Three colonies were inoculated into MHB (Oxoid), and the bacterial suspension was adjusted to a turbidity equivalent to a 0.5 McFarland standard (approximately 10^5^–10^6^ colony-forming units (CFU)/mL) using sterile normal saline. Minimum inhibitory concentrations (MICs) were determined in 96-well microtiter plates. Each plate included positive growth control wells (containing MHB and bacterial inoculum without an antimicrobial agent) and negative sterility control wells (containing MHB only). Plates were incubated at 37 °C for 24 h. All determinations were performed in triplicate on separate occasions, and *E. coli* ATCC 25922 was included as a reference strain. The MIC was defined as the lowest concentration of antimicrobial agent that completely inhibited visible bacterial growth, as assessed by unaided visual inspection. Antimicrobial agents were purchased from MeilunBio (Dalian, China) and are listed in Table 1.

### 2.5. Genomic DNA Sequencing and Analysis

The DNA of each ESBL-producing *E. coli* isolate was extracted using a TIANamp Bacteria DNA Kit (Tiangen Biotech, Beijing, China) according to the manufacturer’s instructions. Sequencing was performed using an Illumina Nextera XT library with 2 × 300 bp paired-end reads (BGI, Shenzhen, China), yielding an average of 2,232,425 reads per isolate (63× average coverage). Raw data were assembled using SPAdes (version 3.0). Multilocus sequence types (MLST), plasmid replicon types, serotypes, virulence genes, and antimicrobial resistance genes were identified using MLST 2.0, PlasmidFinder 2.0, SeroTyperFinder 2.0, VirulenceFinder 2.0, and ResFinder 3.0, respectively, all available from the Center for Genomic Epidemiology database (http://genomicepidemiology.org/, accessed on 15 January 2026). Plasmids were also analyzed using PLACNETw (https://castillo.dicom.unican.es/upload/, accessed on 26 January 2026).

Parsnp v2.0 was used to align the core genome of ESBL-producing *E. coli* isolates, to call single-nucleotide polymorphisms (SNPs), and to generate a core-genome SNP tree with 1000 bootstrap resamples [17].

### 2.6. Conjugation Experiments

Before conjugation experiments, ESBL-producing *E. coli* isolates were tested for susceptibility to sodium azide. Conjugation experiments were then performed using sodium azide-resistant *E. coli* J53 as a recipient by filter mating as described previously [18].

The transferability of ESBL-encoding plasmids was assessed by filter mating using a sodium azide-resistant derivative of *E. coli* J53 as the recipient strain. Donor isolates (ESBL-producing isolates) were cultivated in MHB (Oxoid) supplemented with 2,6-diaminopimelic acid (DAP, 50 µg/mL) (Merck, Shanghai, China). DAP is an essential component of the peptidoglycan layer in certain bacterial species and was included here because preliminary experiments indicated that several of our *E. coli* donor strains exhibited impaired growth or loss of viability in standard MHB (Oxoid), potentially due to cell wall synthetic defects associated with their clinical origin. The addition of DAP restored robust growth and ensured consistent donor cell integrity during the mating procedure. Donor and recipient strains were grown to the mid-logarithmic phase (OD_600nm_ 0.5–0.6), and equal volumes of each culture were mixed. The mixture was collected on a sterile 0.45 µm nitrocellulose filter (Merck, Shanghai, China) placed on an MHA (Oxoid) plate supplemented with DAP (50 µg/mL, Merck) and incubated overnight at 37 °C. Following incubation, the filter was resuspended in sterile saline (Tiangen Biotech, Beijing, China), and serial dilutions were plated onto MHA (Oxoid) containing azide (100 µg/mL, Merck, Shanghai, China) plus cefotaxime (2 μg/mL, MeilunBio) for each plasmid to enumerate transconjugants. Simultaneously, donor counts were determined on MHA (Oxoid) containing DAP (50 µg/mL, Merck) and cefotaxime (2 μg/mL, MeilunBio), and recipient counts were confirmed on MHA (Oxoid) containing azide (100 µg/mL) alone. Conjugation frequency was calculated for each successful mating as the number of transconjugants (CFU/mL) divided by the number of donor cells (CFU/mL) at the end of the mating period.

## 3. Results

### 3.1. Antimicrobial Resistance

Among 276 *E. coli* isolates from buffaloes with subclinical mastitis, 54 were identified as ESBL-producing *E. coli.* All isolates were subjected to antimicrobial susceptibility testing (Table 2). Among the ESBL-producing *E. coli* isolates, all demonstrated resistance to ampicillin and cefotaxime, followed by resistance to cephalothin (75.9%), trimethoprim-sulfamethoxazole (75.9%), ceftiofur (70.4%), florfenicol (68.5%), and cefazolin (63.0%). Lower resistance rates were observed for colistin (40.7%), enrofloxacin (33.3%), gentamicin (25.9%), amoxicillin–clavulanic acid (20.4%), amikacin (1.9%), and imipenem (1.9%). Among non-ESBL-producing *E. coli* isolates, the highest resistance rate was observed for trimethoprim–sulfamethoxazole (74.0%), followed by enrofloxacin (37.8%), doxycycline (35.1%), florfenicol (34.2%), gentamicin (30.6%), and colistin (23.4%). Lower resistance rates were observed for amoxicillin–clavulanic acid (13.5%), cephalothin (9.9%), ampicillin (9.0%), cefazolin (7.7%), cefotaxime (6.3%), ceftiofur (5.4%), amikacin (0.9%), and imipenem (0%).

ESBL-producing isolates exhibited markedly elevated MICs to β-lactams compared with non-ESBL-producing isolates (Table 3). The MIC_50_ and MIC_90_ of ampicillin were 8 and 16 µg/mL, respectively, in ESBL producers, versus 0.06 and 0.25 µg/mL in non-ESBL producers. For cefotaxime, the MIC_50_ was 256 µg/mL (compared with 4 µg/mL), and the MIC_90_ was 512 µg/mL (compared with 64 µg/mL). Both the MIC_50_ and MIC_90_ of ceftiofur were 16 µg/mL in ESBL-producing isolates, compared with 0.12 and 0.5 µg/mL in non-ESBL-producing isolates. Cephalothin MIC_50_ and MIC_90_ values (64 and 128 µg/mL) were 16- and 8-fold higher, respectively, than those in non-ESBL producers (4 and 16 µg/mL). MICs of amoxicillin–clavulanic acid and imipenem were comparable between the two groups. The MIC_50_ of enrofloxacin was identical (0.12 µg/mL) across ESBL and non-ESBL isolates, whereas the MIC_90_ was higher among ESBL producers (8 vs. 2 µg/mL). The MIC_50_ of florfenicol was twofold higher in ESBL producers. Doxycycline MIC_50_ and MIC_90_ values were higher in ESBL producers, while the MIC_90_ of colistin sulfate was lower (32 vs. 64 µg/mL).

### 3.2. Phylogenetic Groups, MLST, and Serotypes of ESBL-Producing E. coli

The predominant phylogroup was group B1 (23/54, 42.6%), followed by A (16/54, 29.6%) and D (14/54, 25.9%), whereas only one ESBL-producing isolate belonged to phylogroup B2. MLST analysis of 54 ESBL-producing *E. coli* isolates identified ST50 as the most frequent sequence type (ST) at 20.4% (11/54), followed by ST2797 (11.1%, 6/54). ST398 was also identified (3.7%, 2/54). The isolates were associated with serogroups O150 (70.4%, 38/54), O28 (18.5%, 10/54), O154 (1.0%, 1/54), O8 (1.9%, 1/54), O162 (1.9%, 1/54), O89 (1.9%, 1/54), and O173 (1.85%, 1/54).

### 3.3. Virulence Gene Distribution

**Figure 1:**
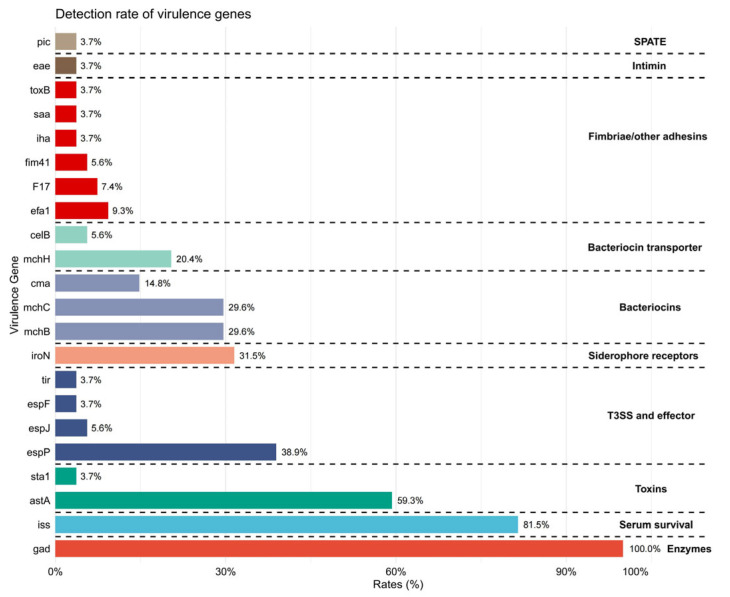
Distribution of virulence genes among ESBL-producing *E. coli* isolates.

Among the 54 ESBL-producing *E. coli* isolates, *iss*, *astA*, *espP*, and *iroN* were detected at 81.5% (44/54), 59.3% (32/54), 38.9% (21/54), and 31.5% (17/54), respectively (Figure 1). Other virulence genes, such as *cma*, *mchB/C*, and *mchH*, showed positive rates ranging from 14.8% (8/54) to 29.6% (16/54), while other virulence genes were rarely detected.

### 3.4. ESBL Gene Distribution

**Figure 2:**
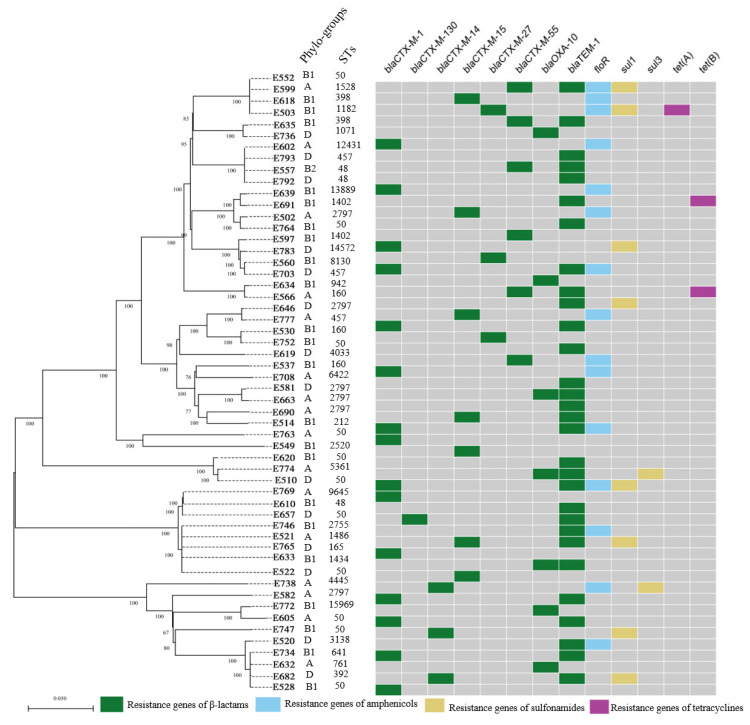
The core-genome single-nucleotide polymorphism (SNP) tree of 54 ESBL-producing *E. coli* isolates. The tree was generated using Parsnp with 1000 bootstrap resamples. Phylogroups, sequence types (STs), and serogroups are indicated. The scale bar represents the number of SNP substitutions per site.

ESBL-encoding genes were detected in all isolates, with *bla_CTX-M_* and *bla_OXA_* types present (Figure 2). The most prevalent ESBL gene was blaCTX-M-1 (27.8%, 15/54), followed by blaCTX-M-15 (13.0%, 7/54). Other ESBL genes detected included *bla_CTX-M-55_* (11.1%, 6/54), *bla_CTX-M-27_* (1.8%, 1/54), *bla_CTX-M-14_* (5.6%, 3/54), and *bla_CTX-M-130_* (1.8%, 1/54). In addition to these ESBL genes, other narrow-spectrum β-lactamase genes were also detected, including *bla_TEM-1_* (55.56%, 30/54) and *bla_OXA-10_* (14.8%, 7/54).

### 3.5. Characteristics of ESBL Gene-Carrying Plasmids

Bioinformatic analysis predicted that all ESBL genes were associated with plasmid-derived contigs, which ranged in size from approximately 32 to 187 kb. Based on in silico prediction and conjugation assays, all plasmids appeared to be transferable via conjugation, with observed conjugation frequencies varying between 4.8 × 10^−7^ and 4.1 × 10^−2^ (Table 3). Putative plasmid replicon types identified among contigs harboring ESBL genes included IncFIB (10/54), IncFIA (10/54), IncI1 (8/54), IncFIA + I1 (5/54), and IncFII (4/54). Additionally, these ESBL-carrying plasmid-derived contigs were predicted to carry additional antimicrobial resistance genes, including those conferring resistance to chloramphenicol (*floR*), sulfonamides (*sul1* and *sul3*), tetracyclines (*tet*(A) and *tet*(B)), and trimethoprim (*dfrA1* and *dfrA12*) (Table 4).

## 4. Discussion

In this study, we determined the antimicrobial susceptibility, distribution of virulence and antimicrobial resistance genes, and plasmid characteristics of ESBL-producing *E. coli* isolates recovered from buffalo mastitis milk in China. Our results indicate that 19.6% (54/276) of the isolates were ESBL-producing *E. coli*. All ESBL producers were resistant to ampicillin and cefotaxime and also exhibited high resistance rates to cephalothin, trimethoprim–sulfamethoxazole, ceftiofur, florfenicol, and cefazolin. Phylogroup B1 was predominant, followed by phylogroups A and D. Isolates showed diverse STs and serotypes. Virulence genes, including *iss*, *astA*, *espP*, and *iroN*, were carried by most ESBL-producing isolates, and *bla_CTX-M_* was the predominant ESBL gene type. Based on in silico analysis of short-read sequencing data, all the detected ESBL genes were predicted to be located on plasmids, which were inferred to belong mainly to IncFIB, IncFIA, IncI1, IncFIA + I1, and IncFII replicon types. Additional resistance genes (*floR*, *sul1*, *sul3*, *tet*(*A*), and *tet*(*B*)) were also predicted to be present on these plasmids. To the best of our knowledge, this study provides insights into ESBL-producing *E. coli* from buffalo mastitis, which is important for ensuring buffalo health and welfare.

*E. coli* remains one of the most significant etiological agents of bovine mastitis. Classical studies have established that *E. coli* predominantly infects the mammary gland during the periparturient and early lactation periods, characteristically inducing acute, local clinical mastitis [19]. However, the role of *E. coli* in subclinical mastitis has also been increasingly documented across diverse geographic settings. In Portugal, *E. coli* was identified as the second most prevalent bacterial species, following coagulase-negative staphylococci (CNS), in bulk tank milk [20]. Similarly, in Uruguay, *E. coli* ranked second only to *S. aureus* among pathogens isolated from bovine subclinical mastitis cases [21]. In China, *E. coli* was reported as one of the predominant coliform bacteria recovered from the milk of cows with subclinical mastitis [22,23]. Collectively, these findings underscore the dual clinical manifestation of *E. coli* infections, ranging from classic acute presentations to persistent subclinical states, thereby reinforcing the relevance of its inclusion in the present study.

ESBL-producing *E. coli* isolates from mastitis milk have raised global concern for both veterinary and public health [24,25]. In this study, the occurrence of ESBL-producing *E. coli* from mastitic buffaloes (19.6%) was lower than that reported in a previous study from China [26]. ESBL-producing *E. coli* isolates showed resistance to a variety of antimicrobial agents. Prolonged use of antibiotics, which exerts selective pressure for the emergence and dissemination of resistant isolates, may contribute to this phenomenon [27]. In this study, *bla_CTX-M-15_* (7/54) was the most prevalent genotype, followed by *bla_CTX-M-55_* (6/54). *bla_TEM-1_*, a narrow-spectrum β-lactamase gene, was also carried by most isolates (55.6%), which is consistent with findings in *E. coli* from bovine mastitis milk in China and Brazil [28,29]. This contrasts with a previous report from Nigeria, in which *bla_CTX-M-1_* was the most prevalent gene (56.1%) and also showed the highest rate in *E. coli* from bovine mastitis milk [30]. The variation in the distribution of β-lactamase genes across geographic regions may stem from several factors, such as genetic mobility, plasmid associations, host and geographic dissemination, and selective pressures exerted by antibiotic use [31].

Phylogroups are widely used as predictors of *E. coli* pathogenicity in humans and cattle. Phylogroup B1 is considered the main pathogenic *E. coli* group in cattle and is also the most prevalent group in bovine mastitis worldwide [32]; similar results were observed in the present study. Meanwhile, phylogroup A is believed to be primarily associated with environmental sources. However, up to 29.6% of ESBL-producing *E. coli* isolates in this study belonged to phylogroup A, indicating that environmental contamination of teats with *E. coli* also plays an important role in the development of mastitis in buffaloes. Therefore, appropriate management practices, such as daily waste removal and avoidance of moisture on the farm, may help reduce the risk of contamination and control mastitis in buffaloes. Because phylotyping has limited discriminatory ability for epidemiological source tracking, we performed MLST. A variety of STs was observed, with ST50 being the most prevalent (11/54, 20.4%). However, this ST is rarely reported in both humans and animals. Importantly, because our study did not systematically capture herd-level, farm-level, or temporal structure, it remains unclear whether the predominance of ST50, serotype O150, and phylogroup B1 reflects a broader population-level signal or possible local clonal enrichment within the sampled buffalo populations. Therefore, while these findings identify a potentially distinctive profile, they should not be overinterpreted as representing the general ESBL-producing *E. coli* population in water buffaloes without further large-scale, longitudinal, and multi-farm sampling.

Several factors contribute to antimicrobial resistance in *E. coli* from mastitis milk, including β-lactamase production, target site modifications, and efflux pumps. AMR genes (*tet(A)*, *mcr-1*, and *bla_CTX-M_*) located on plasmids can be transferred horizontally among animals, humans, and the environment [33]. The *bla_CTX-M_* gene has been reported to be mainly located on IncF group plasmids [34]. In this study, *bla_CTX-M_* genes were predicted to be located on a variety of plasmid types, including IncI1, IncFIA, IncHI2, and IncFIB. The conjugation efficiency, as assessed by laboratory mating assays, of IncI1 plasmids appeared to be related to their size. Our results differ from previous reports that indicate that plasmids carrying the *bla_CTX-M-15_* gene mainly belong to IncI1 and often exhibit higher conjugation efficiency compared to other plasmid types [35]. It seems that IncI1 plasmids carrying AMR genes or virulence determinants may have reduced transfer efficiency due to increased metabolic burden on the donor.

A limitation of this study is that plasmid characterization relied exclusively on short-read sequencing data and in silico prediction tools. Therefore, the localization of ESBL genes to plasmids, the reported plasmid sizes, and their Inc-type assignments are putative and require validation by long-read sequencing, hybrid assembly, or S1-PFGE for definitive confirmation.

## 5. Conclusions

In conclusion, this study provides the first comprehensive characterization of ESBL-producing *E. coli* from water buffaloes with subclinical mastitis. The novelty of this work lies in the identification of potentially distinctive phylogenetic (predominant B1 phylogroup, ST50) and serological (O150) profiles, coupled with the co-carriage of multiple clinically relevant resistance genes on presumably transferable IncF plasmids based on in silico prediction and conjugation experiments. However, given that herd-level, farm-level, and temporal structures were not systematically captured in this study, we cannot definitively distinguish whether the observed predominance of ST50/O150/B1 reflects a broader population-level signal or local clonal enrichment. Consequently, these findings should be interpreted as hypothesis-generating, and further studies incorporating longitudinal, multi-farm sampling are needed to confirm whether these profiles are truly characteristic of ESBL-producing *E. coli* in water buffalo populations. These findings suggest that water buffaloes may represent previously underrecognized reservoirs of clinically relevant, multidrug-resistant ESBL-producing *E. coli* capable of horizontal gene transfer, raising the concern that buffalo farming systems could contribute to the dissemination of such strains. From a practical standpoint, the identification of predominant sequence types and plasmid types circulating in buffalo populations provides actionable molecular targets for the design of farm-level surveillance programs and rapid diagnostic screening tools. Furthermore, the observed transferability of ESBL-encoding plasmids under laboratory conditions underscores the risk of resistance dissemination within mixed-species livestock environments and highlights the urgent need for antimicrobial stewardship interventions—such as the restriction of critically important antimicrobials and the implementation of routine susceptibility testing in buffalo farming systems. These findings also lay the groundwork for future risk assessment studies aimed at quantifying the potential for zoonotic transmission along the dairy production chain.

## Figures and Tables

**Table 1:** Clinical breakpoints of the antimicrobial agents tested in this study.

Antimicrobial Agents	Breakpoints	Range (mg/L)	
		ESBL	Non-ESBL
Ampicillin	≤0.25, 0.5, 1≥	0.125–32	0.03–16
Amoxicillin–clavulanic acid	≤0.25/0.12, 0.5/0.25, 1.0/0.5≥	0.125–32	0.03–16
Ceftiofur	≤2, 4, 8≥	0.06–32	0.03–16
Cephalothin	≤8, 16, 32≥	0.5–128	0.03–16
Cefotaxime	≤8, 16–32, 64≥	1–512	0.5–128
Cefazolin	≤2, 4, 8≥	0.25–128	0.125–64
Enrofloxacin	≤0.12, 0.25, 0.5≥	0.03–16	0.03–16
Gentamicin	≤2, 4, 8≥	0.25–128	0.25–128
Amikacin	≤4, 8, 16≥	0.03–16	0.03–16
Florfenicol	≤4, 8, 16≥	0.125–64	0.03–16
Trimethoprim–sulfamethoxazole	≤2/38, -, 4/76≥	0.06/2.38–32/608	0.06/2.38–32/608
Doxycycline	≤0.12, 0.25, 0.5≥	0.03–16	0.03–16
Colistin sulphate	≤1, 2≥	0.06–32	0.06–32
Imipenem	≤1, 2, 4≥	0.015–8	0.015–8

**Table 2:** Antimicrobial susceptibility testing for ESBL-producing and non-ESBL-producing *E. coli* isolates.

Antimicrobial Agents	Range (mg/L)		Resistant Rates (%)	
	ESBL	Non-ESBL	ESBL	Non-ESBL
Ampicillin	16 to >64	0.03 to 16	100%	9.01%
Amoxicillin–clavulanic acid	0.03/0.02 to 4/2	0.03/0.02 to 4/2	20.37%	13.51%
Ceftiofur	0.06 to >32	0.03 to 16	70.37%	5.41%
Cephalothin	1 to 128	0.03 to 16	75.93%	9.91%
Cefotaxime	64 to 512	2 to 128	100%	6.31%
Cefazolin	0.25 to 64	0.12 to 32	62.96%	7.66%
Enrofloxacin	0.03 to 16	0.03 to 4	33.33%	37.83%
Gentamicin	0.5 to 64	0.5 to 32	25.93%	30.63%
Amikacin	0.03 to 16	0.03 to 16	1.85%	0.9%
Florfenicol	0.12 to >64	0.06 to >16	68.52%	34.23%
Trimethoprim–sulfamethoxazole	0.12/4.75 to >32/608	0.06/2.38 to 32/608	75.93%	73.97%
Doxycycline	0.03 to 16	0.03 to 8	72.22%	35.14%
Colistin sulfate	0.12 to 64	0.06 to 32	40.74%	23.42%
Imipenem	0.03 to 1	0.03 to 1	1.85%	0

**Table 3:** Comparison of MIC_50_ and MIC_90_ between ESBL-producing and non-ESBL-producing *E. coli* isolates.

Antimicrobial Agents	MIC_50_		MIC_90_	
	ESBL	Non-ESBL	ESBL	Non-ESBL
Ampicillin	8	0.06	16	0.25
Amoxicillin–clavulanic acid	0.12/0.06	0.06/0.03	1/0.5	1/0.5
Ceftiofur	16	0.12	16	0.5
Cephalothin	64	4	128	16
Cefotaxime	256	4	512	64
Cefazolin	16	2	32	16
Enrofloxacin	0.12	0.12	8	2
Gentamicin	1	1	32	32
Amikacin	0.5	0.5	2	1
Florfenicol	8	4	64	64
Trimethoprim–sulfamethoxazole	16/304	16/304	32/608	32/608
Doxycycline	2	0.25	16	8
Colistin sulphate	1	0.5	32	64
Imipenem	0.25	0.25	0.5	0.5

**Table 4:** Characteristics of ESBL-producing *E. coli* isolates and their *bla_ESBL_* gene-carrying plasmids.

Isolates	Size (kb)	Inc Group	Conjugation Efficiency
E502	133	HI2	5.6 × 10^−7^
E503	85	FIA + I1	1.4 × 10^−5^
E510	157	FIB	6.2 × 10^−6^
E514	97	I1	4.8 × 10^−4^
E520	36	F	2.5 × 10^−2^
E521	73	FIA	6.3 × 10^−4^
E522	106	I1	5.2 × 10^−5^
E528	108	FII	6.4 × 10^−6^
E530	69	HI2	8.1 × 10^−5^
E537	82	FIA	7.2 × 10^−4^
E549	139	I1	1.5 × 10^−6^
E552	58	FIA + I1	3.9 × 10^−4^
E557	89	FIB + HI2	7.4 × 10^−5^
E560	43	FIB	4.8 × 10^−3^
E566	44	FIA	3.2 × 10^−4^
E581	110	FIB + Q1	4.9 × 10^−6^
E582	109	FIA + I1	9.3 × 10^−6^
E597	32	FIB	1.7 × 10^−2^
E599	187	FII	6.7 × 10^−6^
E602	47	I1	3.8 × 10^−4^
E605	105	FIB	4.2 × 10^−4^
E610	65	X1	7.2 × 10^−5^
E618	38	FIB	9.6 × 10^−3^
E619	117	HI2	7.6 × 10^−5^
E620	53	FIC	6.7 × 10^−4^
E632	139	FIB	5.2 × 10^−6^
E633	58	FII	1.7 × 10^−4^
E634	83	FII	3.6 × 10^−5^
E635	74	I1	5.3 × 10^−4^
E639	58	FIB + FII	3.7 × 10^−4^
E646	184	FIA + HI2	4.8 × 10^−7^
E657	85	FIB	6.8 × 10^−4^
E663	165	I1	6.7 × 10^−6^
E682	40	FIA	2.7 × 10^−3^
E690	64	FIA	2.6 × 10^−4^
E691	42	X2	1.6 × 10^−3^
E703	98	FIA	2.8 × 10^−5^
E708	38	X1	4.1 × 10^−2^
E734	73	FIA	7.3 × 10^−4^
E736	173	HI2	7.5 × 10^−7^
E738	150	FIB	1.3 × 10^−6^
E746	41	X1	1.4 × 10^−3^
E747	169	FIA	7.4 × 10^−6^
E752	36	I1	2.6 × 10^−2^
E763	94	FIA	9.5 × 10^−4^
E764	103	FIB + I1	6.4 × 10^−5^
E765	73	FIA	9.3 × 10^−5^
E769	63	FIB	8.3 × 10^−4^
E772	85	FIA + I1	4.6 × 10^−5^
E774	53	I1	1.3 × 10^−3^
E777	68	FIB	6.8 × 10^−4^
E783	48	FIA + I1	3.6 × 10^−3^
E792	147	FIA + FIB	3.7 × 10^−6^
E793	56	HI2	2.1 × 10^−4^

## Data Availability

The data presented in this study are openly available in GenBank at NCBI, reference number PRJNA1450891.
